# Varietal dataset of nutritionally important *Lablab purpureus* (L.) Sweet from Eastern Uttar Pradesh, India

**DOI:** 10.1016/j.dib.2019.103935

**Published:** 2019-04-19

**Authors:** Ajeet Singh, P.C. Abhilash

**Affiliations:** Institute of Environment & Sustainable Development, Banaras Hindu University, Varanasi, India

**Keywords:** Agrobiodiversity, Distribution maps, Food security, *Lablab purpureus*, Legumes, Morphological traits, Eastern Uttar Pradesh, Varietal dataset

## Abstract

Legumes are one of the important crops for food and nutritional security. According to the International Treaty on Plant Genetic Resources for Food and Agriculture, the collection and documentation of promising germplasms are essential for creating the global database and also for facilitating the global exchange for crop improvement and further exploitation. Presented here are varietal dataset of an agriculturally important legume, *Lablab purpureus* (L.) Sweet, collected from eastern Uttar Pradesh of North India. Extensive field surveys were conducted for studying the occurrence and distribution of *L. purpureus* in six districts of eastern Uttar Pradesh (Ballia, Ghazipur, Jaunpur, Mirzapur, Sonebhadra and Varanasi) and germplasms of promising varieties were collected, and cultivated for further characterization. Dataset provides the morphological traits such as variation in stem colour, leaf size, flower colour, pod colour, pod size, seed size, seed weight etc. of fourteen different varieties of *L. purpureus* grown in the field gene bank maintained by authors at Rajgarh block of Mirzapur district, eastern Uttar Pradesh, India. Additionally, national and global distribution maps of *L. purpureus* was prepared using ArcGIS platform.

**Table:** Specifications table

Subject area	*Agricultural Sciences, Environmental Sciences*
More specific subject area	*Agrobiodiversity, Agronomy, Crop Science*
Type of data	*Table, figure, distribution map*
How data was acquired	*Direct observation through field survey and experiment. The data regarding the global and regional distribution of Lablab purpureus (L.) Sweet was collected from the literature and distribution maps were developed using ArcGIS Desktop 10 (ESRI, Redlands, California, USA), ESRIs ArcMap™ 10.0 (Build 2414) for windows program. The cluster grouping of L. purpureus was done using SPSS for windows version 16.0 (SPSS Inc., Chicago, USA)*
Data format	*Raw and analysed primary data*
Experimental factors	*Random survey followed by field visit, collection, cultivation and field validation*
Experimental features	*Standard agronomic practices followed for the cultivation of selected fourteen varieties and filed data were obtained periodically*
Data source location	*Varanasi, India, Institute of Environment & Sustainable development, BHU, Varanasi*
Data accessibility	*Data is with this article*
Related research article	*P. Vidigal, B. Durate, A.R. Cavaco, I. Cacador, A. Figueiredo, A.R. Matos, W. Viegas, F. Monteiro, Preliminary diversity assessment of an undervalued tropical bean (Lablab purpureus (L.) Sweet) through fatty acid profiling, Plant Physiol. Biochem., 132, 2018, 508–514*[1]*.*

**Table:** 

**Value of the Data** • *Lablab purpureus* (L.) Sweet is a nutritionally significant legume for human and animal consumption. • Varietal dataset is important for maintaining the global database of such important species and also for crop breeding and agro-biodiversity conservation. • National and global distribution maps are imperative for framing national and global conservation initiatives. • Dataset will serve as a source of information to various stakeholders across the world, regarding crop diversification by intercropping with *L. purpureus.*

## 1 Data

Sustainable crop production for meeting the food and nutritional requirements of a rapidly growing human population is one of the major humanitarian crisis for this twenty first century and therefore, the creation of dataset regarding the occurrence, distribution and varietal diversity of nutritionally relevant crops are paramount important for framing suitable conservation measure and also for national and global food security [2], [3], [4]. In this context, the present study provides the varietal dataset of a nutritionally significant legume species. *Lablab purpureus* (L.) Sweet is an ancient legume species cultivated throughout in Asia and African countries for food and nutritional security [1], [2], [3], [4]. The dataset presented here is a national (Fig 1A) global distribution map of *L. purpureus* (Fig. 1B) and morphological traits of fourteen different varieties i.e. AS-PCA-Lp (1); AS-PCA-Lp (2); AS-PCA-Lp (3); AS-PCA-Lp (4); AS-PCA-Lp (5); AS-PCA-Lp (6); AS-PCA-Lp (7); AS-PCA-Lp (8); AS-PCA-Lp (9); AS-PCA-Lp (10); AS-PCA-Lp (11); AS-PCA-Lp (12); AS-PCA-Lp (13); and AS-PCA-Lp (14), collected from six districts of eastern Uttar Pradesh (Ballia, Ghazipur, Jaunpur, Mirzapur, Sonebhadra and Varanasi districts), north India. The details of surveyed sites are shown in Table (1). The colour plates showing the varietal diversity of *L. purpureus* is presented in Fig. (2), whereas the morphological variations in pods of *L. purpureus* such as colour, length, width, shape etc. is presented in Fig (3) and cluster grouping of *L. purpureus* based on pod length and pod width is presented in Fig. (4). Similarly, the diversity in seed size and shape of mature seeds and dried seeds are presented in Fig. (5) and Fig. (6), respectively. Table (2A) shows the qualitative morphological traits (stem colour, leaf vein colour, flower colour and pod colour) whereas Table (2B) shows the quantitative morphological traits (Leaflet length, leafltet width, petiole length, pod width, fresh pod weight of three pods, number of seeds per pod, fresh seed weight of hundred seeds, mature seed length and mature seed width) of fourteen different *L. purpureus* varieties [i.e. AS-PCA-Lp (1) to AS-PCA-Lp (14)] cultivated in the field gene bank of *L. purpureus* maintained by authors at Rajgarh, Mirzapur district of eastern Uttar Pradesh., India.

**Fig. 1 (A):**
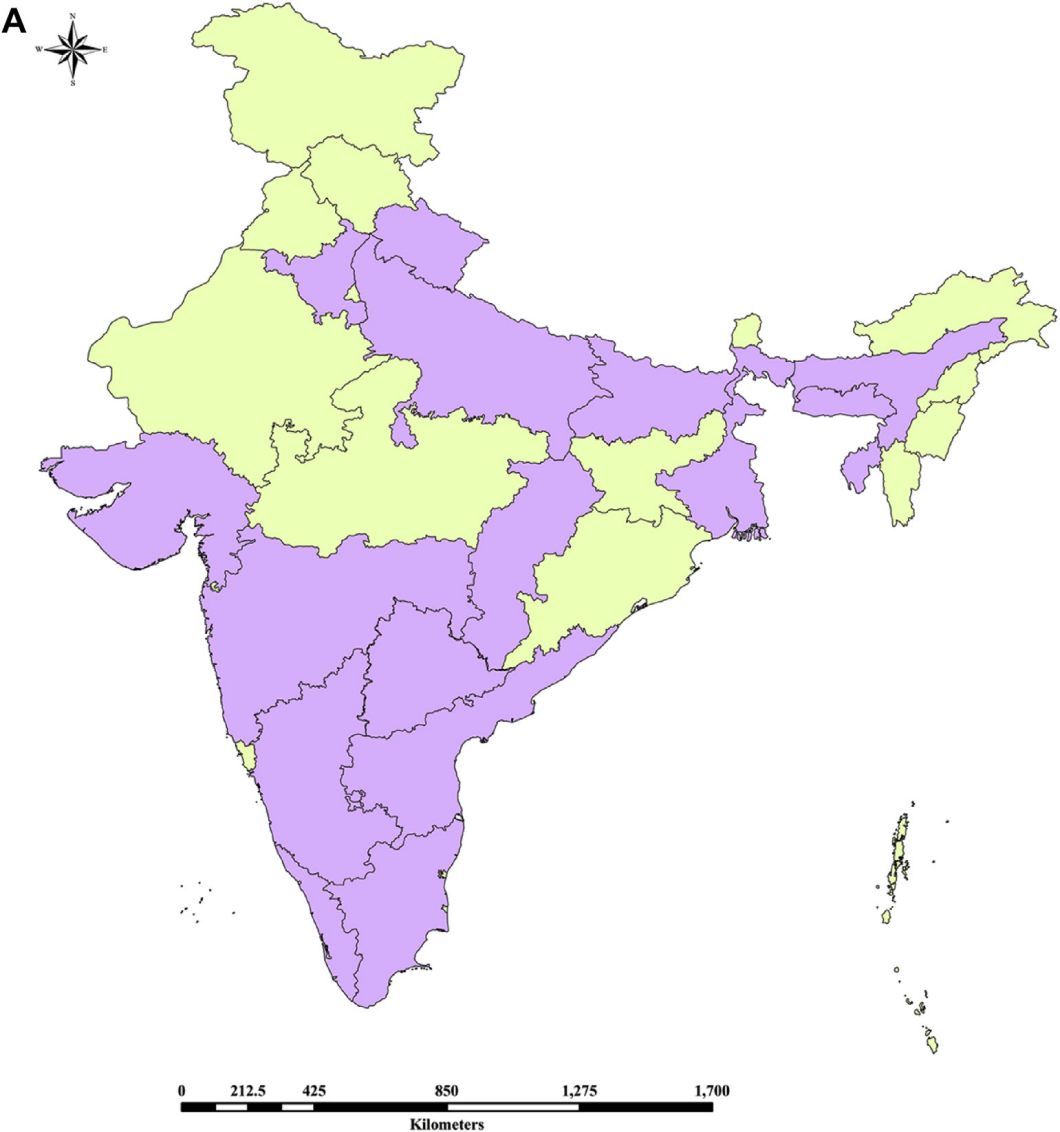
National distribution map of *Lablab purpureus* (L.) Sweet, prepared on the basis information gathered from literature [11], [12], [13], [14], [15], [16], database (www.lablab.org) as well as from direct field visit. The light purple colour shows the distribution range in India.

**Fig. 1 (B):**
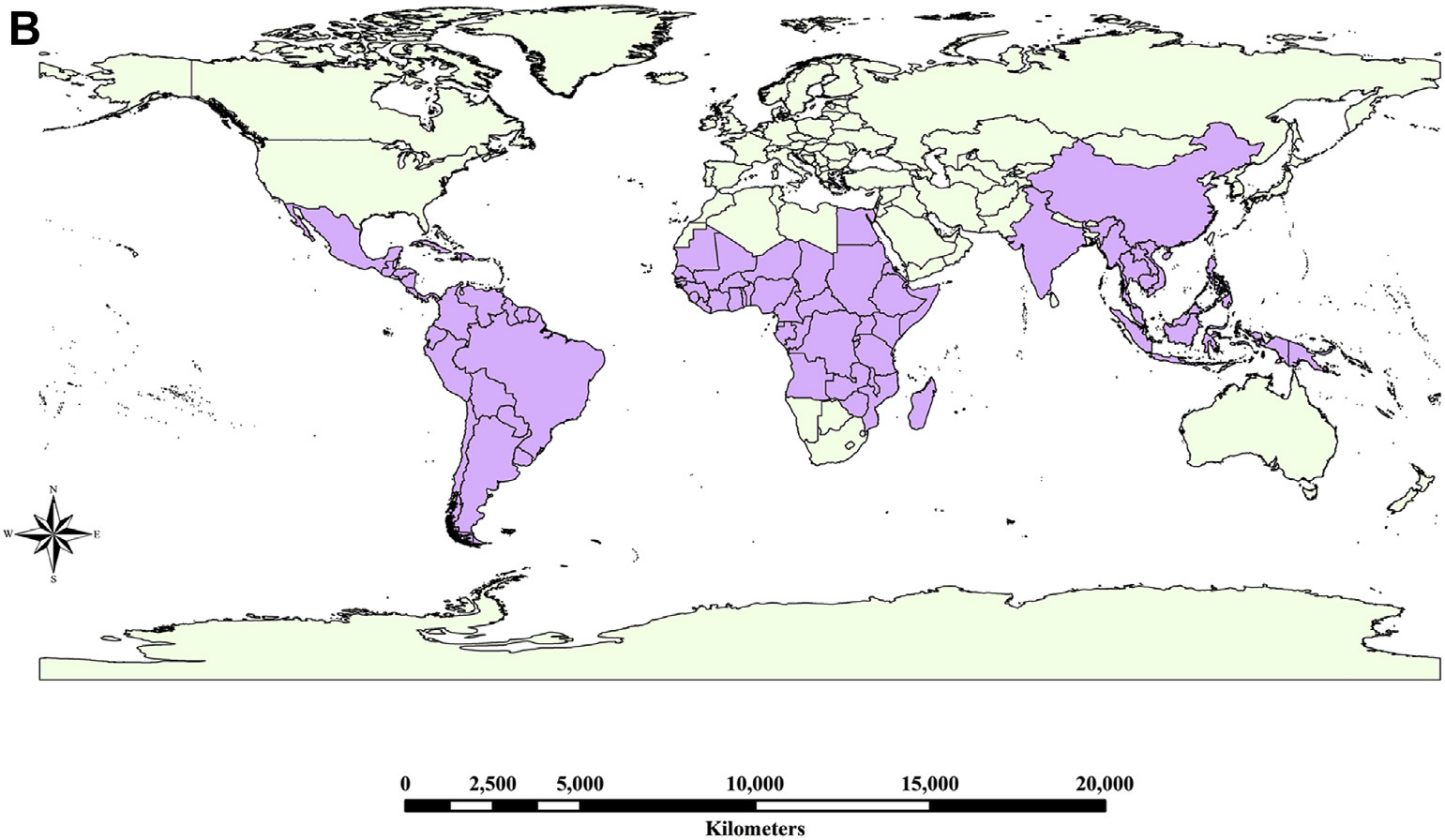
Global distribution map of *Lablab purpureus* (L.) Sweet was prepared using ArcGIS platform on the basis of information collected from literature [1], [5], [6], [7], [8], [9], [10] as well as from the databases of international agencies such as FAO, Wild Crop Relatives, Bioversity International and International Legume Database and Information Service (ILDIS). The light purple colour shows the global distribution range.

**Table 1:** Details of field survey conducted for recording the distribution as well as collecting promising varieties of *Lablab purpureus* (L.) Sweet germplasms for further characterization.

District^a^	Location^a^	Latitude and Longitude	Abundance^b^	Habitat
Mirzapur (n = 48)	Kailhut (n = 18)	25°09′16.5″N, 82°56′51.6″E	++	Railway track
	Barevan (n = 11)	25°07′45.8″N, 82°55′43.0″E	+++	Near pond side
	Bakiyabad (n = 5)	25°06′26.9″N, 82°53′41.4″E	++	Road side
	Kon (n = 14)	24°50′35.6″N, 82°52′22.4″E	++	Pond side
Sonebhadra (n = 33)	Chopan (n = 9)	24°31′31.0″N, 83°02′02.5″E	++	Degraded land
	Salkhan (n = 4)	24°33′39.5″N, 83°02′24.8″E	+++	Road side
	Piparwar (n = 5)	24°53′02.6″N, 82°53′53.2″E	+++	Kitchen garden
	Maraipur (n = 4)	24°52′22.6″N, 82°55′14.4″E	++	Near pond
	Renukoot (n = 6)	24°12′34.8″N, 83°02′17.3″E	++	Degraded site
	Obra (n = 5)	24°27′25.2″N, 83°00′51.8″E	+++	Pond area
Varanasi (n = 27)	Dinapur (n = 11)	25°21′04.2″N, 83°03′12.2″E	+++	Road side
	Basani (n = 6)	25°26′38.8″N, 82°49′45.3″E	++	Field area
	Dafi (n = 5)	25°14′38.9″N 82°58′42.9″E	++	Backyard garden
	Sarai dangari (n = 5)	25°13′39.3″N 82°58′38.3″E	+++	Boundary wall
Ballia (n = 13)	Nawada (n = 3)	25°49′40.4″N, 84°00′43.0″E	+	Road side
	Mithanpur (n = 2)	25°49′23.8″N, 84°00′35.7″E	+	Field area
	Bansdih (n = 2)	25°52′51.4″N, 84°13′06.5″E	++	Field area
	Ghosi road (n = 3)	25°59′37.5″N, 83°49′53.9″E	+	Road side
	Bhadikara (n = 3)	26°02′08.2″N, 84°02′43.6″E	++	Field area
Jaunpur (n = 16)	Tarapur (n = 8)	25°44′30.2″N, 82°40′09.1″E	+++	Kitchen garden
	Muradganj (n = 5)	25°44′31.8″N, 82°39′53.5″E	++	Water lodging site
	Budhkarpur (n = 3)	25°45′33.9″N, 82°41′33.9″E	+++	Field area
Ghazipur (n = 11)	Kalauta (n = 3)	25°33′56.4″N, 83°32′16.4″E	++	Farmers field
	Bakuliapur (n = 3)	25°35′57.8″N, 83°34′10.1″E	++	Boundary wall
	Tulasipur (n = 2)	25°34′33.7″N, 83°32′14.8″E	+	Road side
	Mugalani chak (n = 1)	25°35′00.9″N, 83°32′56.3″E	+++	Kitchen garden
	Sukhadeopur (n = 2)	25°35′48.4″N, 83°35′56.3″E	+	Near railway track

a The number in parenthesis is the number of villagers surveyed in the region.

b **Abundance:** High (+++), medium (++), and low (+).

**Fig. 2:**
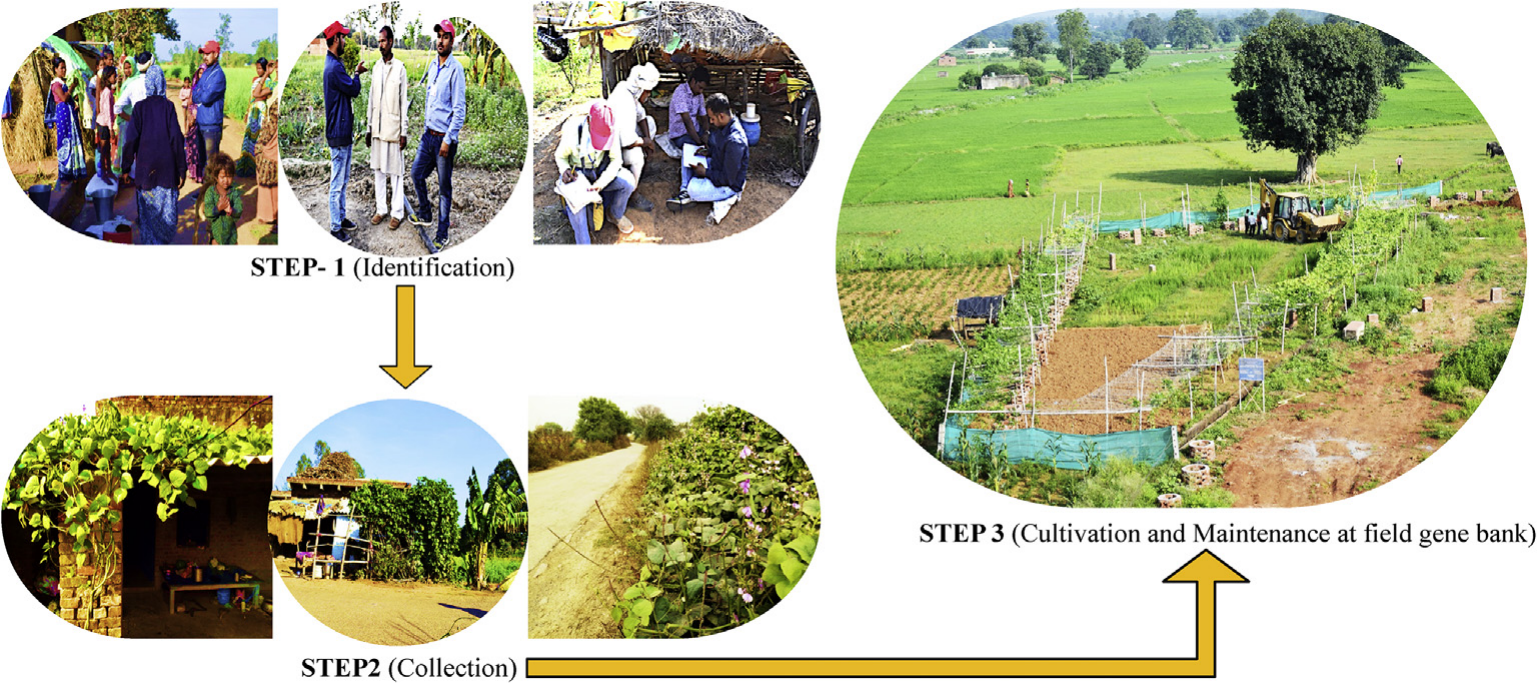
The overall approach employed for the distribution of *Lablab purpureus* (L.) Sweet in Eastern Uttar Pradesh, India and various steps involved in the creation of field gene bank of promising *L. purpureus* varieties. These varieties were collected from diverse habitat such as kitchen garden/backyard garden, road side, pond side, disturbed side and other geographical areas of Eastern Uttar Pradesh and field gene bank of these varieties is maintained at Rajgarh block of Mirzapur District of Eastern Uttar Pradesh, India.

**Fig. 3:**
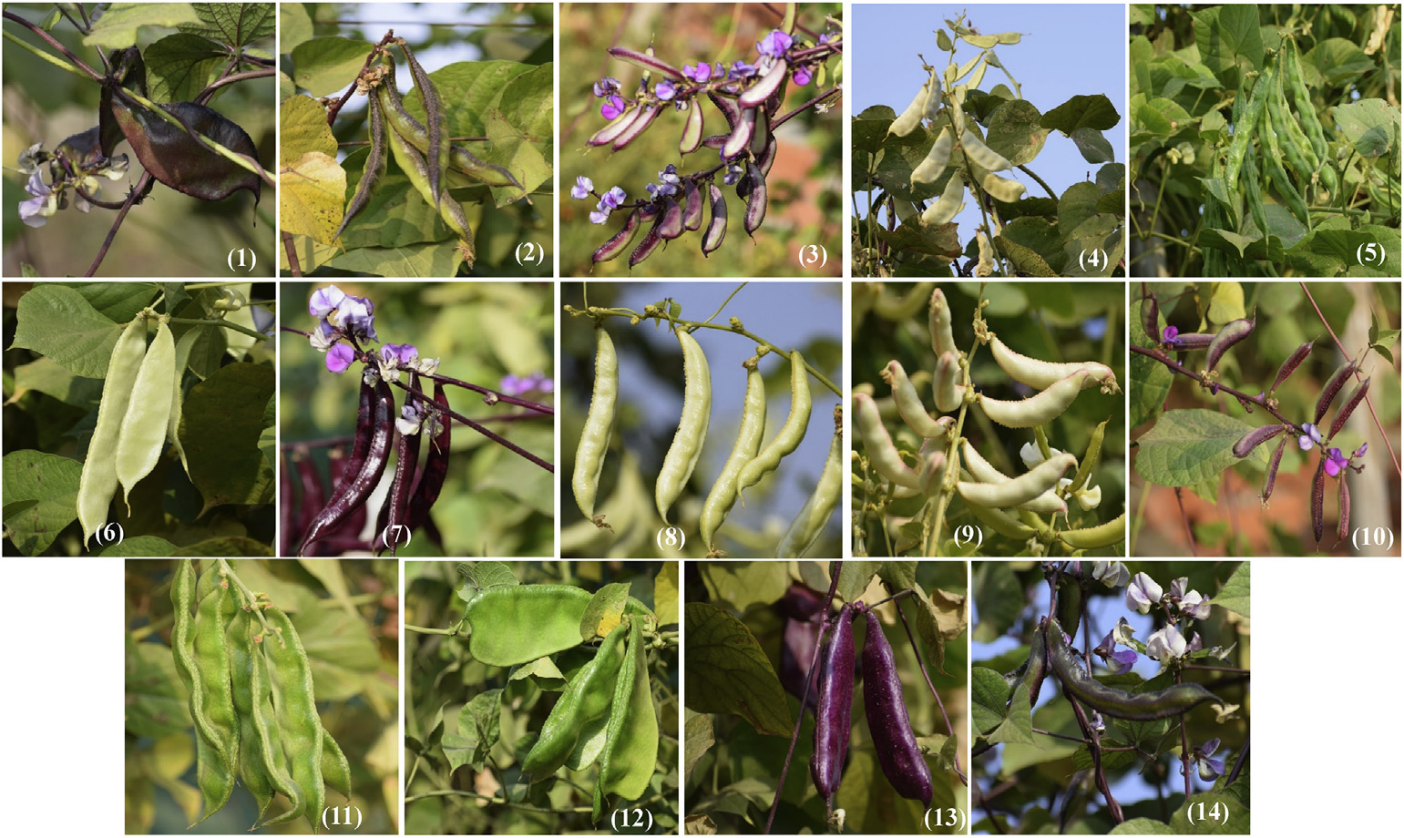
Varietal Diversity of *Lablab purpureus* (L.) Sweet in Eastern Uttar Pradesh, India. Plate no (1) to (14) represents different varieties i.e. AS-PCA-Lp (1); AS-PCA-Lp (2); AS-PCA-Lp (3); AS-PCA-Lp (4); AS-PCA-Lp (5); AS-PCA-Lp (6); AS-PCA-Lp (7); AS-PCA-Lp (8); AS-PCA-Lp (9); AS-PCA-Lp (10); AS-PCA-Lp (11); AS-PCA-Lp (12); AS-PCA-Lp (13); and AS-PCA-Lp (14). These varieties were collected from diverse habitat such as kitchen garden/backyard garden, road side, pond side, disturbed side and other geographical areas of Eastern Uttar Pradesh and field gene bank of these varieties is maintained at Rajgarh block of Mirzapur District of Eastern Uttar Pradesh, India.

**Fig. 4:**
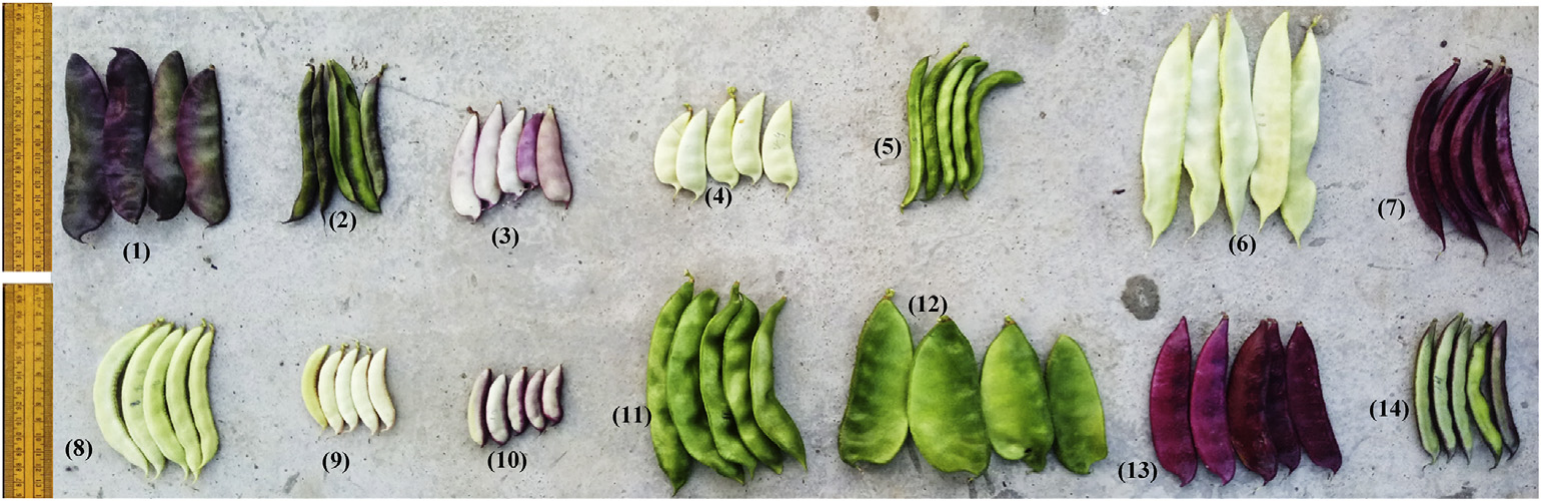
Diversity of pods size, shape and color in *Lablab purpureus* (L.) Sweet harvested during the mature stage. Plate no (1) to (14) represents different varieties i.e. AS-PCA-Lp (1); AS-PCA-Lp (2); AS-PCA-Lp (3); AS-PCA-Lp (4); AS-PCA-Lp (5); AS-PCA-Lp (6); AS-PCA-Lp (7); AS-PCA-Lp (8); AS-PCA-Lp (9); AS-PCA-Lp (10); AS-PCA-Lp (11); AS-PCA-Lp (12); AS-PCA-Lp (13); and AS-PCA-Lp (14). These varieties were collected from diverse habitat such as kitchen garden/backyard garden, road side, pond side, disturbed side and other geographical areas of Eastern Uttar Pradesh and field gene bank of these varieties is maintained at Rajgarh block of Mirzapur District of Eastern Uttar Pradesh, India.

**Fig. 5:**
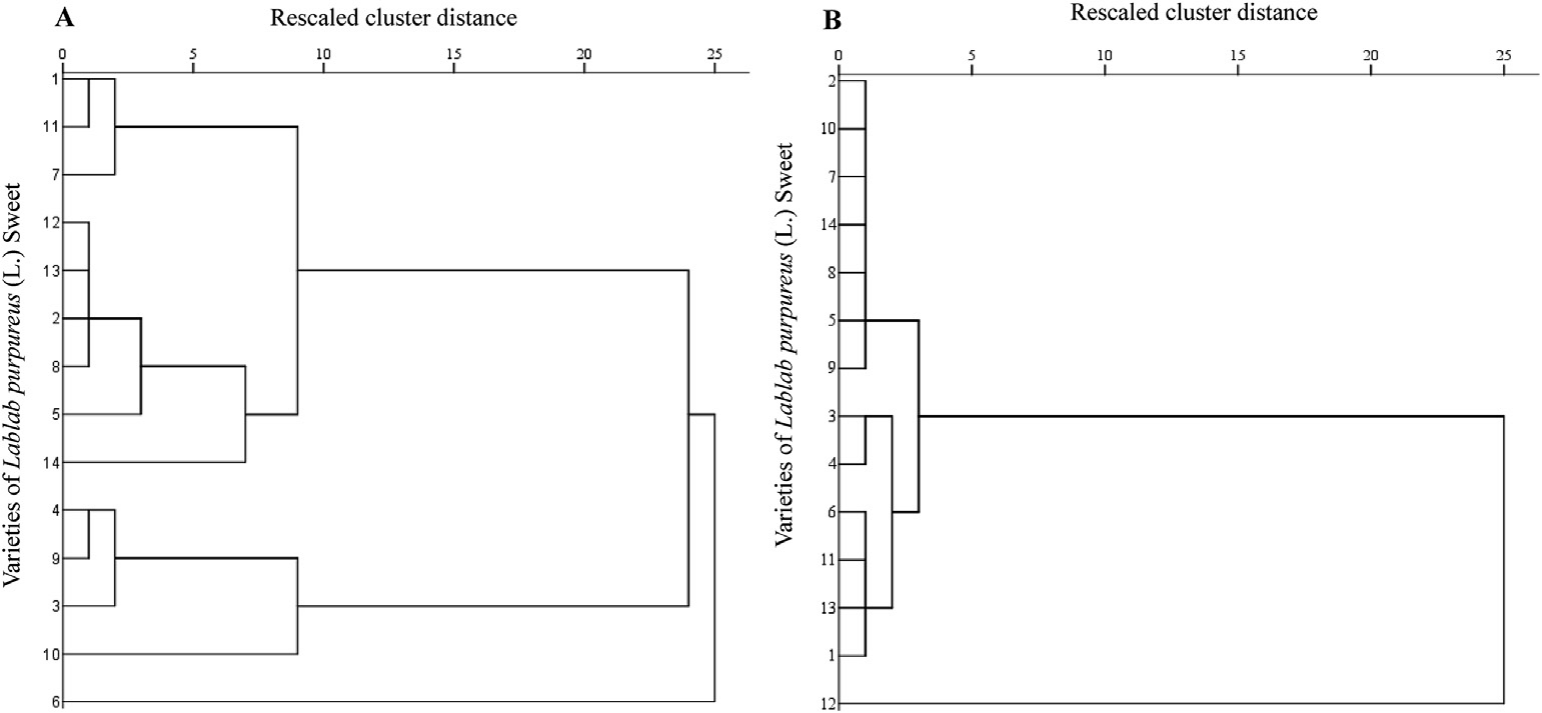
Cluster grouping of *Lablab purpureus* (L.) Sweet, based on (A) pod length and (B) pod width were done using SPSS (version 16.0) for windows program. The clustering showed quite variation in pod length whereas not much variation in pod width. Variety no (1) to (14) represents different varieties such as AS-PCA-Lp (1); AS-PCA-Lp (2); AS-PCA-Lp (3); AS-PCA-Lp (4); AS-PCA-Lp (5); AS-PCA-Lp (6); AS-PCA-Lp (7); AS-PCA-Lp (8); AS-PCA-Lp (9); AS-PCA-Lp (10); AS-PCA-Lp (11); AS-PCA-Lp (12); AS-PCA-Lp (13); and AS-PCA-Lp (14). These varieties were collected from diverse habitat such as kitchen garden/backyard garden, road side, pond side, disturbed side and other geographical areas of Eastern Uttar Pradesh and field gene bank of these varieties is maintained at Rajgarh block of Mirzapur District of Eastern Uttar Pradesh, India.

**Fig. 6:**
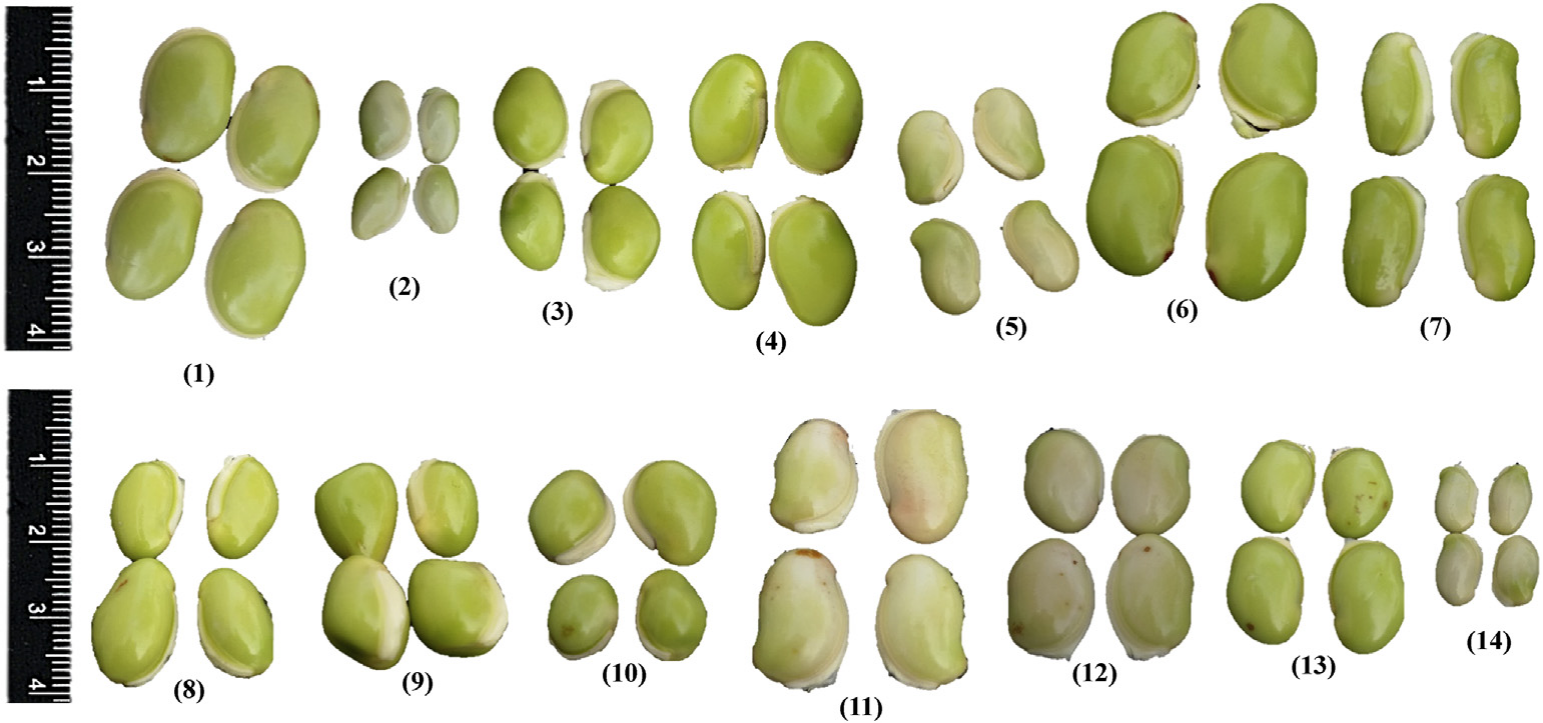
Diversity in seed size of *Lablab purpureus* (L.) Sweet harvested during the mature stage. Plate no (1) to (14) represents different varieties i.e. AS-PCA-Lp (1); AS-PCA-Lp (2); AS-PCA-Lp (3); AS-PCA-Lp (4); AS-PCA-Lp (5); AS-PCA-Lp (6); AS-PCA-Lp (7); AS-PCA-Lp (8); AS-PCA-Lp (9); AS-PCA-Lp (10); AS-PCA-Lp (11); AS-PCA-Lp (12); AS-PCA-Lp (13); and AS-PCA-Lp (14). These varieties were collected from diverse habitat such as kitchen garden/backyard garden, road side, pond side, disturbed side and other geographical areas of Eastern Uttar Pradesh and field gene bank of these varieties is maintained at Rajgarh block of Mirzapur District of Eastern Uttar Pradesh, India.

**Table 2A:** Morphological traits (qualitative traits) of 14 varieties of *Lablabpurpureus* grown in the field gene bank maintained by authors at Rajgarh, Mirzapur, UP, India.

Varietal ID	Stem colour	Leaf vein colour	Flower colour	Pod colour
AS-PCA-Lp (1)	Purple	Purple	Pink	Greenish purple
AS-PCA-Lp (2)	Purple	Green	Purple	Green with violet edges
AS-PCA-Lp (3)	Dark pink	Pink	Pink	White with violet edges
AS-PCA-Lp (4)	Green	Green	White	Light green
AS-PCA-Lp (5)	Green	Green	White	Green
AS-PCA-Lp (6)	Light green	Green	White	Whitish green
AS-PCA-Lp (7)	Dark red	Dark red	Purple	Dark red
AS-PCA-Lp (8)	Light green	Light green	White	Whitish green
AS-PCA-Lp (9)	Light green	Light green	White	White with pink edges
AS-PCA-Lp (10)	Red	Light red	Purple	White with purple edges
AS-PCA-Lp (11)	Green	Light green	White	Green
AS-PCA-Lp (12)	Green	Green	White	Green
AS-PCA-Lp (13)	Dark red	Red	Purple	Dark red
AS-PCA-Lp (14)	Dark red	Dark red	Pink	Green with purple edges

**Table 2B:** Morphological traits (quantitative traits) of 14 varieties of Lablab *purpureus* grown in the field gene bank maintained by authors at Rajgarh, Mirzapur, UP, India.

Varietal ID	Leaflet length (cm)	Leaflet width (cm)	Petiole length (cm)	Pod length (cm)	Pod width (cm)	Fresh pod weight (3 pods) (g)	No of Seeds Per Pod	Fresh seed weight (100 seeds) (g)	Mature seed length (cm)	Mature seed width (cm)
AS-PCA-Lp (1)	6.86 ± 2.32	6.11 ± 1.76	6.41 ± 2.77	13.5 ± 1.32	3.17 ± 0.04	15.61 ± 0.32	4.66 ± 0.57	158.55 ± 1.92	1.66 ± 0.05	1.16 ± 0.05
AS-PCA-Lp (2)	7.21 ± 1.63	6.13 ± 2.32	8.66 ± 2.75	11.16 ± 0.76	1.33 ± 0.41	5.81 ± 0.26	5.33 ± 0.57	40.95 ± 0.83	1.26 ± 0.11	0.76 ± 0.05
AS-PCA-Lp (3)	7.23 ± 2.25	5.76 ± 2.15	5.93 ± 2.11	7.00 ± 1.56	2.16 ± 0.32	7.72 ± 0.86	4.00 ± 1.23	99.09 ± 1.59	1.23 ± 0.15	0.96 ± 0.11
AS-PCA-Lp (4)	6.16 ± 2.75	5.26 ± 2.10	6.83 ± 3.25	6.03 ± 0.95	2.11 ± 0.11	6.04 ± 0.29	6.00 ± 1.13	71.01 ± 1.15	1.76 ± 0.05	1.06 ± 0.05
AS-PCA-Lp (5)	6.73 ± 2.75	5.83 ± 2.10	7.83 ± 3.25	10.16 ± 0.95	1.03 ± 0.11	12.23 ± 1.02	6.33 ± 1.01	51.93 ± 1.15	1.06 ± 0.05	0.66 ± 0.05
AS-PCA-Lp (6)	9.10 ± 2.54	9.20 ± 2.07	13.66 ± 4.80	15.66 ± 1.04	2.8 ± 0.20	31.54 ± 1.21	6.00 ± 0.57	39.00 ± 1.89	1.46 ± 0.15	0.90 ± 0.05
AS-PCA-Lp (7)	8.66 ± 1.96	7.81 ± 1.47	13.33 ± 5.03	12.83 ± 1.89	1.36 ± 0.11	20.76 ± 1.19	6.00 ± 1.14	31.84 ± 0.93	1.13 ± 0.05	0.56 ± 0.02
AS-PCA-Lp (8)	7.93 ± 3.01	7.00 ± 2.62	10.33 ± 7.37	10.83 ± 1.75	1.51 ± 040	31.07 ± 0.86	6.00 ± 1.21	60.31 ± 0.61	1.33 ± 0.05	0.46 ± 0.05
AS-PCA-Lp (9)	6.51 ± 1.80	5.23 ± 1.12	8.43 ± 2.13	6.43 ± 0.40	0.70 ± 0.10	9.54 ± 0.63	4.00 ± 1.10	30.95 ± 0.99	1.16 ± 0.05	0.70 ± 0.12
AS-PCA-Lp (10)	5.76 ± 1.55	5.21 ± 1.57	7.83 ± 3.32	4.76 ± 0.58	1.31 ± 0.51	8.65 ± 0.42	4.00 ± 1.14	52.35 ± 1.37	1.36 ± 0.06	1.13 ± 0.05
AS-PCA-Lp (11)	6.96 ± 1.67	6.33 ± 1.92	11.83 ± 4.31	13.41 ± 0.52	2.91 ± 0.11	35.87 ± 0.93	5.66 ± 0.57	71.41 ± 0.96	1.23 ± 0.15	1.16 ± 0.11
AS-PCA-Lp (12)	6.8 6 ± 1.55	6.96 ± 1.66	10.26 ± 5.32	11.43 ± 1.25	5.03 ± 0.68	51.54 ± 1.02	4.66 ± 0.57	61.58 ± 0.90	1.53 ± 0.11	1.21 ± 0.11
AS-PCA-Lp (13)	7.02 ± 1.32	6.52 ± 1.13	13.33 ± 6.65	11.61 ± 0.72	2.66 ± 0.15	30.38 ± 1.17	5.66 ± 0.57	52.30 ± 0.95	1.51 ± 0.27	1.03 ± 0.15
AS-PCA-Lp (14)	6.16 ± 1.12	5.66 ± 1.32	6.26 ± 1.10	9.11 ± 0.85	1.16 ± 0.32	10.73 ± 0.82	5.31 ± 0.96	25.08 ± 0.61	0.93 ± 0.15	0.66 ± 0.05

## 2 Experimental design, materials, and methods

The data regarding the global distribution of *L. purpureus* was collected from published papers and also from international database for wild crops such as FAO (www.unfao.org), Tropical Forages (www.tropicalforage.info), Crop Wild Relatives (www.cwrdiversity.org), Biodiversity International (www.bioversityinternational.org), International Legume Database and Information Services (www.ildis.org) etc. and the distribution maps were developed using ArcGIS Desktop 10 (ESRI, Redlands, California, USA), ESRIs ArcMap™ 10.0 (Build 2414) for windows program. (Fig. 1 (A), Fig. 1 (B)A and 1B). Similarly, the distribution of *L. purpureus* in India was prepared based on the literature survey as well as direct field visit (Fig. 1B). The varietal dataset of *L. purpureus* presented here was obtained through three different steps such as (i) exploration of *L. purpureus* in eastern Uttar Pradesh (ii) Collection and characterization of promising germplasms and (iii) cultivation of promising species at the field gene bank for data collection (Fig. 2). Additionally, cluster grouping of *L. purpureus* varieties were done according to their pod length and pod width.

### 2.1 Exploration of L. purpureus in Eastern Uttar Pradesh

Extensive field surveys were conducted in selected districts of eastern Uttar Pradesh (Ballia, Ghazipur, Jaunpur, Mirzapur, Sonebhadra and Varanasi districts), India and information regarding the cultivation, usage and current status of *L. purpureus* in the study area was gathered through structured questionnaire survey (Table 1). For this, 148 farmers were interviewed and identified the current cultivation localities/areas of *L. purpureus* and promising varieties were collected for characterization and further evaluation. The varieties were collected from diverse habitat such as kitchen garden/backyard garden, road side, pond side, disturbed side and other geographical areas of Eastern Uttar Pradesh and field gene bank of these varieties is maintained at Rajgarh block of Mirzapur District of Eastern Uttar Pradesh, India for further characterization and germplasm maintenance.

### 2.2 Collection and characterization of promising germplasms

As mentioned earlier, 14 promising varieties of *L. purpureus* named AS-PCA-Lp (1); AS-PCA-Lp (2); AS-PCA-Lp (3); AS-PCA-Lp (4); AS-PCA-Lp (5); AS-PCA-Lp (6); AS-PCA-Lp (7); AS-PCA-Lp (8); AS-PCA-Lp (9); AS-PCA-Lp (10); AS-PCA-Lp (11); AS-PCA-Lp (12); AS-PCA-Lp (13); and AS-PCA-Lp (14) were selected for cultivating at the field gene bank (Fig. 3) for further characterization and standard agronomic practices including spacing pattern irrigation, manuring, crop diversification etc. were optimized for large-scale cultivation.

### 2.3 Cultivation of promising species and data collection

**Fig. 7:**
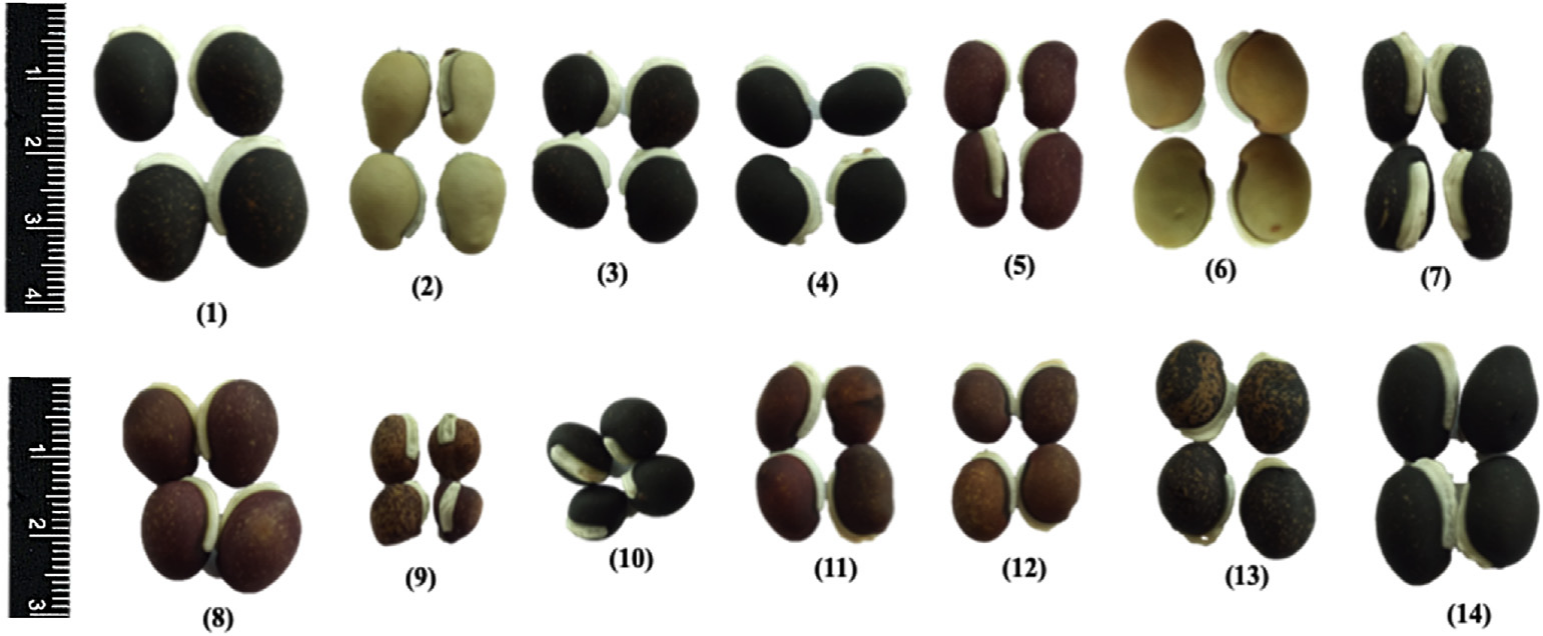
Diversity in dried seed size of *Lablab purpureus* (L.) Sweet. Plate no (1) to (14) represents different varieties i.e. AS-PCA-Lp (1); AS-PCA-Lp (2); AS-PCA-Lp (3); AS-PCA-Lp (4); AS-PCA-Lp (5); AS-PCA-Lp (6); AS-PCA-Lp (7); AS-PCA-Lp (8); AS-PCA-Lp (9); AS-PCA-Lp (10); AS-PCA-Lp (11); AS-PCA-Lp (12); AS-PCA-Lp (13); and AS-PCA-Lp (14). These varieties were collected from diverse habitat such as kitchen garden/backyard garden, road side, pond side, disturbed side and other geographical areas of Eastern Uttar Pradesh and field gene bank of these varieties is maintained at Rajgarh block of Mirzapur District of Eastern Uttar Pradesh, India.

Selected varieties of *L. purpureus* were cultivated at the field gene bank for obtaining morphological traits. Standard agronomic practices were employed and varietal traits such as stem colour, leaf size, flower colour, pod length (Fig. 4), pod width (Fig. 5), seed size of mature seeds (Fig. 6), dried seeds (Fig. 7) etc. were obtained for each and every varieties (Table 2A, Table 2BB). The data were presented as mean value ± standard deviation. The cluster grouping of *L. purpureus* was done using SPSS (version 16.0) for windows program (SPSS Inc., Chicago, USA).
