# Supplementary material for: Varietal dataset of nutritionally important Lablab purpureus (L.) Sweet from Eastern Uttar Pradesh, India
Source: Data Brief. 2019 Apr 19;24:103935. doi: 10.1016/j.dib.2019.103935 (PMC6488767; doi:10.1016/j.dib.2019.103935)
Supplement: Supplementary file 1 — Multimedia component 1 [file mmc1.docx]

**Conflict of interest**

Authors do not have any conflict of interest
